# Establishing trimester-specific reference intervals of vitamin K-dependent protein C, protein S, and antithrombin and exploring the impacts of abnormal anticoagulation tests on adverse perinatal complications or outcomes

**DOI:** 10.3389/fgwh.2026.1793289

**Published:** 2026-07-14

**Authors:** Lei Li, Zhengwen Xu, Jiazi Zeng, Hongyuan Zhu, Lin Liu, Yifei Chen, Jingnan Zhang, Huihui Yan, Jingyi Niu, Yue Zhang, Shaofei Su, Yanhong Zhai, Zheng Cao

**Affiliations:** 1 Department of Laboratory Medicine, Beijing Obstetrics and Gynecology Hospital, Capital Medical University, Beijing Maternal and Child Health Care Hospital, Beijing, China; 2 Center of Clinical Mass Spectrometry, Beijing Obstetrics and Gynecology Hospital, Capital Medical University, Beijing Maternal and Child Health Care Hospital, Beijing, China; 3 Department of Obstetrics, Beijing Obstetrics and Gynecology Hospital, Capital Medical University, Beijing Maternal and Child Health Care Hospital, Beijing, China; 4 Department of Ophthalmology, Beijing Xuanwu Traditional Chinese Medicine Hospital, Beijing, China; 5 Information Center, Beijing Obstetrics and Gynecology Hospital, Capital Medical University, Beijing Maternal and Child Health Care Hospital, Beijing, China; 6 Central Laboratory, Beijing Obstetrics and Gynecology Hospital, Capital Medical University, Beijing Maternal and Child Health Care Hospital, Beijing, China

**Keywords:** adverse outcome, antithrombin, pregnancy, protein C, protein S, reference interval

## Abstract

**Background:**

Owing to complex physiological changes that take place in the anticoagulation system during pregnancy, reference intervals (RIs) based on non-pregnant women are not optimal for clinical diagnosis and treatment. In this cohort study, we aimed to establish trimester-specific RIs of vitamin K-dependent protein C (PC), protein S (PS), and antithrombin (AT) in singleton pregnancies and assess the risks of developing adverse pregnancy outcomes.

**Methods:**

Using the nonparametric approach, the first- (*n* = 276), second- (*n* = 124), and third- (*n* = 182) trimester PC, PS, and AT testing results of healthy singleton pregnant women from January 2017 and December 2020 were included to establish the trimester-specific RIs. The retrospective associations between the anticoagulation tests of singleton pregnant women (*n* = 6,090) and pregnancy complications as well as perinatal outcomes were assessed by logistic regression analysis.

**Results:**

The trimester-specific RIs for the PS and AT showed decreasing trends over the entire pregnancy (*p* < 0.05), which was closely associated with metabolism and changes in demand of vitamin K. However, the level of PC was slightly increased from the first to the second trimester and dropped in the third trimester (*p* < 0.05). In the subsequent risk analysis, AT displayed negative associations between its plasma level and the odds of intrahepatic cholestasis of pregnancy, fetal growth restriction, and preterm birth.

**Conclusion:**

With the established trimester-specific RIs of vitamin K-dependent PC, PS, and AT, our study sheds light on the potential application of these tests in identifying women with increased risks of developing various pregnancy complications and adverse postpartum outcomes.

## Background

Pregnancy is associated with remarkable changes in hemostasis (1), resulting in a hypercoagulable state and causing nearly a 5-fold increased risk of venous thromboembolism (VTE) (2). Meanwhile, the physiological anticoagulant system plays a critical role in safely balancing the maternal risk of thromboembolism and hemorrhage, in which vitamin K-dependent protein C (PC) is a key component of this intrinsic pathway (3). Activated protein C functions by downregulating the activity of the coagulation system via cleaving and inhibiting coagulation cofactors FVIIIa and FVa (4). Protein S (PS) is a vitamin K-dependent plasma glycoprotein that circulates in plasma and has multiple anticoagulant properties (5). PS is also an integral part of the body's endogenous anticoagulant system (6) and is an important cofactor that contributes to the inactivation of several fundamental coagulation factors, such as factors (F) Va and VIIIa (7, 8), FXa (9, 10), and FIXa (11, 12). Sufficient levels of vitamin K are required for the synthesis of proteins C and S (13). Antithrombin (AT) is a thrombosis inhibitor, which has an activation effect on devitalized coagulation factors like FXa and FIXa (14, 15). The aforementioned three circulating plasma markers (PC, PS, and AT) are commonly evaluated and monitored by clinicians to assess the function of the anticoagulant system in their patients. Previous studies have reported that, in pregnancy, the activities of PC, PS, and AT gradually decreased as the gestational age went up (16, 17). This physiological change was believed to be an adaption to the resolution of the hemostatic challenges during delivery. However, it may predispose individuals to adverse pregnancy outcomes such as fetal loss, fetal growth restriction, and gestational hypertension (18–20).

The existing reference intervals (RIs) for PC, PS, and AT are derived from non-pregnant adults and thus are inappropriate for pregnant women; they may compromise relevant diagnosis or even intervention. In this study, we established trimester-specific reference intervals (RIs) with healthy pregnant women. Furthermore, we evaluated the associations between the level changes of these anticoagulant markers and the risks of developing pregnancy complications and/or adverse perinatal outcomes.

## Materials and methods

### Design study and population

This is a cross-sectional study conducted from January 2017 to December 2020, in which a total of 582 healthy singleton pregnant women (aged 21–43) who had a live birth at Beijing Obstetrics and Gynecology Hospital and presented with normal pregnancy test results (i.e., routine blood, urine, and biochemical tests) using the hospital's medical record system were enrolled. The exclusion criteria applied in the selection process were as follows: (i) any tumors or diseases of major organs within 1 year, such as nutritional deﬁciency diseases or documented liver, kidney, or other diseases that might affect the values of selected measurands; (ii) positive urinary protein; (iii) history of hypertension and diabetes before pregnancy; (iv) other maternal and fetal complications including gestational diabetes mellitus, pregestational diabetes mellitus, thyroid dysfunction, pregnancy with chronic hypertension, gestational hypertension, chronic hypertension complicated by preeclampsia, preeclampsia, intrahepatic cholestasis, hemolysis, elevated liver enzymes and low platelets count syndrome (HELLP) syndrome, acute fatty liver of pregnancy, intrauterine fetal death, placental abruption, fetal growth restriction, macrosomia, and preterm birth; (v) infectious diseases such as hepatitis B virus, hepatitis C virus, human immunodeﬁciency virus, and syphilis; (vi) fetal malformations and fetal chromosomal abnormalities; (vii) recent medication, surgery, or other treatments; and (viii) acute trauma or acute or chronic inﬂammation. All samples were collected at the fasting status. Reference intervals were established according to the Clinical and Laboratory Standards Institute (CLSI) EP28-A3c guideline. A minimum of 120 reference individuals was included in each partition for nonparametric estimation of the 2.5th and 97.5th percentiles (21).

For the association study, the singleton pregnant women (*n* = 6,090) registered in Beijing Obstetrics and Gynecology Hospital, Capital Medical University for antenatal check-ups with anticoagulation evaluation testing results (PC, PS, and AT) between January 2017 and December 2020 were included. The following peri- and postpartum complications or adverse outcomes were included in the association analyses: gestational hypertension (GH), gestational diabetes mellitus (GDM), preeclampsia (PE), intrahepatic cholestasis of pregnancy (ICP), preterm birth (PTB), fetal growth restriction (FGR) macrosomia, placental abruption (PA), and postpartum hemorrhage (PPH). The definitions of these diseases and conditions are provided in Supplementary Table S1. Given the different onset time of these pregnancy-related diseases, the risk analyses of GH, GDM, PE, and ICP were assessed using the PC, PS, and AT testing results collected in the first trimester of pregnancy. While in the risk analyses of PTB, FGR, macrosomia, PA, and PPH, the laboratory results of PC, PS, and AT retrieved from all three trimesters were investigated.

### Experimental methods

According to the institutional clinical laboratory's standard operation procedures, venous blood supplemented with 0.11 mmol/L (3.2%) sodium citrate was centrifuged at 3,500 r/min for 10 min to separate plasma, followed by immediate PC, PS, and AT testing. The maternal levels of PC, PS, and AT were assayed on the automated coagulation analyzer ACL TOP 700 (Werfen, US), using the PC assay kit (Instrumentation Laboratory, N0622827, Spain), PS assay kit (Instrumentation Laboratory, N0623057, Spain), and AT assay kit (Instrumentation Laboratory, N0522624, Spain), respectively. The intra- assay CV of PC, PS, and AT were 2.5%–3.0%, 5.2%–9.7%, and 3.2%–9.6%, respectively; the inter-assay CV were 3.0%–4.2%, 6.2%–9.4%, and 5.2%–8.8%, respectively.

### Statistical analysis

The Kolmogorov–Smirnov test was used to evaluate the normality of the data distribution. The mean and standard deviation (SD) were used for describing a normally distributed set of data, and the median and percentiles were used for non-normally distributed data. We used t-test to compare two normally distributed sets of data and Mann–Whitney *U* and Kruskal–Wallis tests were used to compare results among the same women across different trimesters. A two-tailed *p*-value < 0.05 was considered significant. The RIs for PC, PS, and AT derived from the healthy pregnant women were estimated by the IBM SPSS Statistic 26 using the nonparametric approach. We calculated the reference intervals using the percentile method. For normally distributed data, mean ± 1.96 SD was used to calculate the RIs. For non-normally distributed data, the reference intervals were usually determined by two percentiles, such as the 2.5th percentile and the 97.5th percentile.

The multivariate logistic regression analysis with odds ratios (ORs) and 95% confidence intervals (CIs) were calculated, while maternal age was adjusted as a confounder. The variable of age was included as a covariate in the logistic regression analysis to calculate the resulting OR and *p* values for the adjusted risk relationship between anticoagulation tests and outcomes. In the logistic analysis, the measurements of serum PC, PS, and AT were converted into binary variables according to the established RIs.

## Results

**Figure 1:**
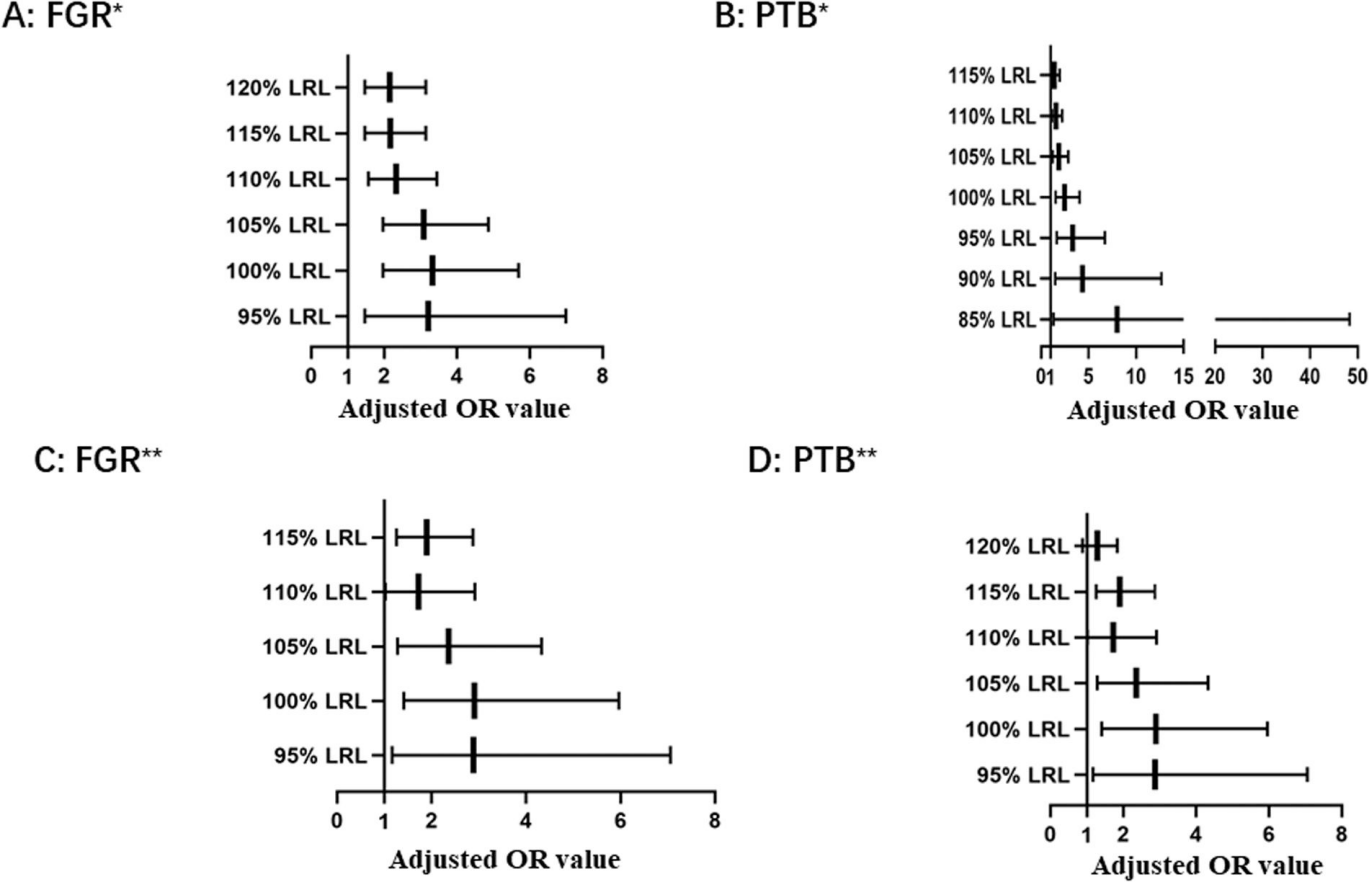
Box plots representing the PC, PS, and AT levels of singleton pregnancies in the first, second, and third trimesters. The levels of PC, PS, and AT in the first (*n* = 276), second (*n* = 124), and third trimesters (*n* = 182); *indicates *p* < 0.05. ns, not statistically significant. PS, protein S; PC, protein C; AT, antithrombin.

The participants included in this study ranged in age from 19 to 51 years old, with an average age of 33.12 ± 4.07 years and a BMI of 23.99 ± 4.27 kg/m^2^. The first- (*n* = 276), second- (*n* = 124), and third- (*n* = 182) trimester PC, PS, and AT testing results of healthy singleton pregnant women were used to establish the trimester-specific RIs. As shown in Figure 1, the levels of PS and AT were significantly lower in the pregnant group and became further decreased as pregnancy progressed, which was in agreement with a hypercoagulation state during pregnancy. Significant activity changes of the PC level were observed among different trimesters. The trimester-specific RIs for the three anticoagulation markers determined with the nonparametric approach were presented in Table 1, showing a similar decreasing trend over the whole pregnancy as indicated in Figure 1.

**Table 1:** Medians and 95% reference intervals for the anticoagulation tests of trimester special pregnant women (*n* = 582).

Indicator	First trimester (*n* = 276)	Second trimester (*n* = 124)	Third trimester (*n* = 182)
PC (%)	103.00 (75.93–152.15)	117.00 (83.00–162.38)	110.00 (71.73–159.00)
PS (%)	44.60 (25.29–78.62)	41.55 (23.80–68.73)	37.30 (20.70–60.88)
AT (%)	99.00 (76.00–121.38)	90.00 (72.13–119.75)	86.00 (62.58–110.43)

**Figure 2:**
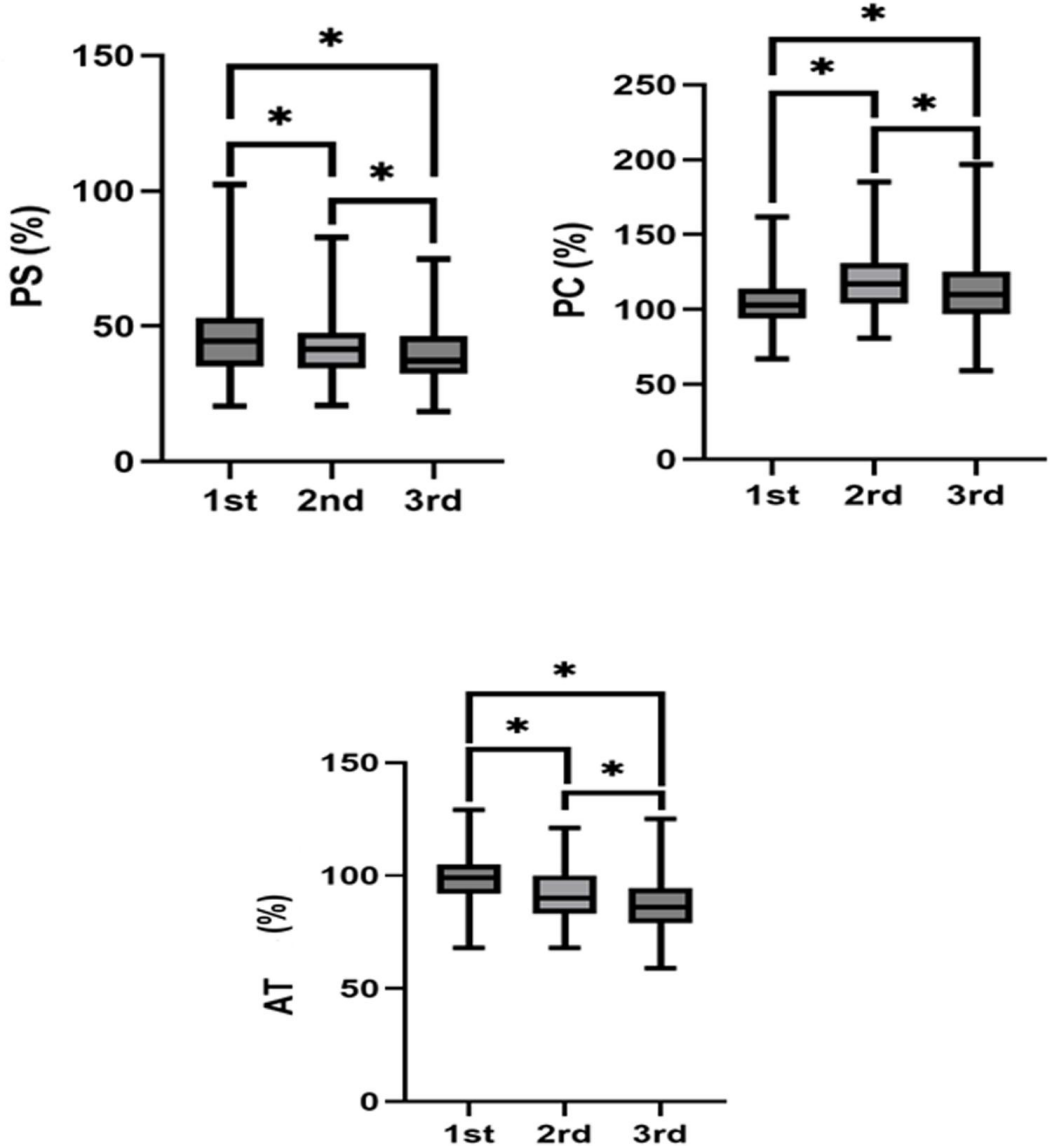
Forest plots of adjusted OR values determined at different AT levels of singleton pregnancy women with FGR or PTB. *: second trimester; **: third trimester; FGR, fetal growth restriction; PTB, preterm birth; LRL, lower reference interval limit, used in the OR analysis and statistically significant (*p* < 0.05).

Among the 6,090 patients, 3,357 were in the early stage, 1,296 were in the middle stage, and 1,437 were in the late stage of pregnancy. Of the patients, 6.85% experienced fetal growth restriction and 11% had premature births. In the logistic regression with age as covariates, the OR values were calculated using the cut-offs set around the lower reference limits (LRLs) of PC, PS, and AT. As shown in Figure 2 and Supplementary Tables S2–S4, AT, not PC or PS, displayed negative associations between plasma level and the risks of developing various pregnancy complications and adverse postpartum outcomes. Specifically, when the second- or third- trimester AT activity was below 95%–120% of its LRL, there was a significant increase of risk for developing FGR (OR = 3.34, 95% CI = 1.96–5.69) and PTB (OR = 7.98, 95% CI = 1.32–48.28) (Figure 2, Supplementary Tables S3, S4). Similarly, decreased AT posed fast-increasing risks (OR_max_ = 51.58) in singleton women with ICP development (Supplementary Table S2).

## Discussion

A deficiency in vitamin K can be devastating for pregnant women and newborns, possibly resulting in hemorrhage (5). In our study of healthy singleton pregnant women, PC remained unchanged in the first trimester but increased in the second trimester, which might relate to neutralizing thrombin generation at the maternal–fetal interface (22). Different from PC, a significant and continual reduction in PS activity was observed throughout the pregnancy, which was in line with previous studies (22, 23). According to a review by Gierula et al. (24), about 40% of PS was present as free PS in the anticoagulation steps. Approximately 60% of PS formed an inactive complex with the complement 4b-binding protein (C4BP), presenting no anticoagulation function. It has been reported that the level of C4BP was increased during pregnancy (25), which may contribute to reduced PS activity in pregnant women. In addition, consistent with James et al. (26), our study showed a downward trend of AT with an increase in gestational age, which may be related to the hypercoagulable state in pregnant women. Pregnancy induced a physiological hypercoagulable state to prevent peripartum hemorrhage. Concentrations and activities of multiple procoagulant factors (Ⅱ, Ⅴ, Ⅶ, Ⅷ, Ⅹ, and fibrinogen) rise markedly, leading to excessive thrombin generation. As the major endogenous anticoagulant, antithrombin is massively consumed to inactivate excess thrombin and activated coagulation factors, resulting in reduced AT activity (26).

In the logistic regression analysis, the level of AT was negatively associated with increased risks of FGR and PTB. As a crucial molecule in the anticoagulation system, the decrease in AT activity may result in placental micro- and/or macro- vascular thrombosis, which in turn increase the risk of placental insufficiency and have a direct negative effect on placentation (27, 28). A dysfunctional placenta can restrain fetal growth and lead to FGR, affecting approximately 10% of all pregnancies (29, 30). In addition, AT has been postulated to present anti-inflammatory properties, which were independent of its function on coagulation (31). Other investigators have shown that AT in circulation could protect leukocytes from premature activation by deactivating leukocyte chemo-attractant receptors (32). As a result, inflammation that was known as a significant risk factor for PTB and was potentially initiated or strengthened by decreased AT activity may eventually contribute to the development of PTB (33).

ICP was the most common pregnancy-induced liver disease and it has a complex etiology with genetic, endocrine, and environmental components (34, 35). In our study, the risk of ICP was associated with decreased AT level, confirming the connections between the two events that have been reported in several earlier retrospective studies (34, 36). These findings support a reasonable speculation that pregnant women with pregnancy-induced antithrombin deficiency may suffer from exaggerated coagulation-fibrinolysis, a decreased interstitial fluid, and decreased circulating plasma volume (37), which, in combination, contribute to the pathogenies of liver dysfunctions such as ICP (38).

In this robust cohort study involving a relatively large patient pool (*n* = 6,090), a few limitations still exist. Firstly, it was a single-center study involving local pregnant women only. The application of the established RIs were limited due to the lack of diverse ethnic and/or social-economic backgrounds (16). Secondly, the long-term association study between the levels of PC, PS, and AT and the incidence of postpartum thrombotic diseases such as VTE were not included due to incomplete follow up records of our patients.

## Conclusion

Here we established trimester-specific RIs of PC, PS, and AT with singleton healthy pregnant women. In addition, decreased AT activity was found to be closely associated with the risks of developing various pregnancy complications and adverse outcomes, proving its potential application in disease warning or prediction in pregnancy.

## Data Availability

The data analyzed in this study is subject to the following licenses/restrictions: According to the patients' verbal consent, their relevant demographic and medical records are only available from the corresponding author on reasonable request. Requests to access these datasets should be directed to Zheng Cao, zhengcao2011@hotmail.com.
