# Supplementary material for: Establishing trimester-specific reference intervals of vitamin K-dependent protein C, protein S, and antithrombin and exploring the impacts of abnormal anticoagulation tests on adverse perinatal complications or outcomes
Source: Front Glob Womens Health. 2026 Jul 14;7:1793289. doi: 10.3389/fgwh.2026.1793289 (PMC13407839; doi:10.3389/fgwh.2026.1793289)
Supplement: Supplementary file 1 [file Table1.docx]

**Supplementary Tables**

| **eTable 1** Diagnosis criteria of pregnancy complications and adverse prenatal outcomes | |  |
| --- | --- | --- |
| Diseases | Definition |  |
|  |  |  |
| GH | Blood pressure elevation [systolic blood pressure≥140 mmHg or diastolic blood pressure+B12≥90 mmHg] at >20 weeks’ gestation in the absence of proteinuria (1). |  |
| GDM | In an oral glucose tolerance test (OGTT), every participant is requested for a 50 g glucose challenge test and serum glucose levels are assayed 1 h later. Subjects with positive results (glucose levels≥7.8 mmol/L) are required to undergo a 75 g OGTT. Serum glucose levels during OGTT are measured at 0, 1 and 2 h, respectively. The normal values are fasting glucose <5.1 mmol/L, 1-h glucose <10.0 mmol/L and 2-h glucose <8.5 mmol/L. GDM is diagnosed if one or more values equaled or exceeded the above thresholds (2). |  |
| PE | New-onset hypertension (systolic blood pressure≥140 mmHg or diastolic blood pressure≥90 mmHg) and new-onset proteinuria (300 mg of protein in 24 h or a urine protein/creatinine ratio of 0.3 mg/dl) after 20 weeks of gestation, in a previously normotensive woman (1). |  |
| ICP | ICP is diagnosed by the presence of abnormal liver function tests and raised maternal serum bile acids. Abnormal liver function tests include elevated levels of alanine aminotransferase, aspartate aminotransferase and/or gamma-glutamyl transpeptidase. The upper limits of total serum bile acids were 10–14 μmol/L in postprandial state and 6–10 μmol/L in fasting state (3). |  |
| PPH | Cumulative blood loss of≥1,000 mL or blood loss accompanied by signs and symptoms of hypovolemia within 24 hours following the birth process (4). |  |
| PTB | Usually defined as regular contractions accompanied by cervical change at less than 37 weeks' gestation (5). |  |
| PA | Defined as premature separation of a normally implanted placenta (6). |  |
| FGR | FGR is defined on the basis of the weight percentile relative to the gestational age. The World Health Organization (WHO) Defines FGR as an estimated fetal weight below the third percentile. However, the American College of Obstetrics and Gynecology (ACOG) defines FGR as an estimated fetal weight below the 10th percentile for gestational age and states that it is frequently associated with placental insufficiency. This is the most commonly used classification (7). |  |
| Macrosomia | Fetal birth weight ≥ 4000 g, regardless of gestational age (8). |  |
| GH: gestational hypertension; GDM: gestational diabetes mellitus; PE: preeclampsia; ICP: intrahepatic cholestasis of pregnancy; PPH: postpartum hemorrhage. PTB: preterm birth; PA: placental abruption; FGR: fetal growth restriction. | |  |
| **References** 1. Hypertension in pregnancy. Report of the American College of Obstetricians and Gynecologists' task force on hypertension in pregnancy. Obstet Gynecol. 2013;122(5):1122–31. 2. World Health Organization. Diagnostic criteria and classification of hyperglycemia first detected in pregnancy: a World Health Organization Guideline. Diabetes Res Clin Pract. 2014;103(3):341–63. 3. Williamson C, Geenes V. Intrahepatic cholestasis of pregnancy. Obstet Gynecol. 2014;124(1):120–33. 4. Practice Bulletin No. 183: Postpartum Hemorrhage. Committee on Practice Bulletins-Obstetrics. Obstet Gynecol. 2017;130(4):e168-e186. 5. Goldenberg RL, Culhane JF, Iams JD, Romero R. Epidemiology and causes of preterm birth. Lancet. 2008 Jan 5;371(9606):75-84.  6. Oyelese Y, Ananth CV. Placental abruption. Obstet Gynecol. 2006 Oct;108(4):1005-16.  7. American College of Obstetricians and Gynecologists' Committee on Practice Bulletins—Obstetrics and the Society for Maternal-FetalMedicin. ACOG Practice Bulletin No. 204: Fetal Growth Restriction. Obstet Gynecol. 2019 Feb;133(2):e97-e109.  8. Macrosomia: ACOG Practice Bulletin, Number 216. Obstet Gynecol. 2020;135(1):e18-e35. | |  |

| **eTable 2** Logistic regression analysis of the risk of PC, PS, and AT in the first trimester during singleton pregnancy for pregnancy complications and adverse perinatal outcomes | | | | | | | | |
| --- | --- | --- | --- | --- | --- | --- | --- | --- |
|  | PC | |  | PS | |  | AT | |
|  | OR (95% CI) | *p* value |  | OR (95% CI) | *p* value |  | OR (95% CI) | *p* value |
| PE^a^ |  |  |  |  |  |  |  |  |
| 80% LRL | 1.65 (0.21-13.29) | 0.64 |  | 0 | 0.98 |  | 4.43 (0.46-42.78) | 0.20 |
| 85% LRL | 2.41 (0.53-10.99) | 0.26 |  | 0.76 (0.18-3.17) | 0.71 |  | 4.43 (0.46-42.78) | 0.20 |
| 90% LRL | 1.93 (0.57-6.53) | 0.29 |  | 0.52 (0.13-2.15) | 0.37 |  | 1.36 (0.17-10.69) | 0.77 |
| 95% LRL | 0.86 (0.27-2.78) | 0.80 |  | 0.50 (0.16-1.59) | 0.24 |  | 1.23 (0.37-4.06) | 0.73 |
| 100% LRL | 0.67 (0.24-1.84) | 0.44 |  | 0.89 (0.41-1.94) | 0.77 |  | 1.68 (0.80-3.55) | 0.17 |
| 105% LRL | 0.52 (0.23-1.20) | 0.13 |  | 0.92 (0.48-1.77) | 0.80 |  | 1.88 (1.14-3.10) | 0.01 |
| 110% LRL | 0.70 (0.39-1.27) | 0.24 |  | 0.86 (0.47-1.57) | 0.62 |  | 1.50 (1.00-2.25) | 0.05 |
| 115% LRL | 0.75 (0.48-1.18) | 0.22 |  | 0.73 (0.41-1.30) | 0.28 |  | 1.31 (0.94-1.83) | 0.12 |
| 120% LRL | 0.85 (0.59-1.22) | 0.37 |  | 0.65 (0.38-1.12) | 0.12 |  | 1.01 (0.74-1.36) | 0.97 |
| GH^a^ |  |  |  |  |  |  |  |  |
| 80% LRL | 0 | 0.98 |  | 3.69 (1.07-12.70) | 0.04 |  | 0 | 0.98 |
| 85% LRL | 0 | 0.98 |  | 2.42 (0.85-6.91) | 0.10 |  | 0 | 0.98 |
| 90% LRL | 0 | 0.98 |  | 2.09 (0.82-5.33) | 0.12 |  | 2.01 (0.26-15.80) | 0.51 |
| 95% LRL | 0.40 (0.06-2.94) | 0.37 |  | 1.88 (0.85-4.16) | 0.12 |  | 1.17 (0.28-4.91) | 0.83 |
| 100% LRL | 0.48 (0.12-1.97) | 0.31 |  | 1.34 (0.61-2.93) | 0.46 |  | 0.85 (0.26-2.73) | 0.78 |
| 105% LRL | 0.64 (0.26-1.59) | 0.34 |  | 0.93 (0.43-2.03) | 0.86 |  | 1.04 (0.50-2.16) | 0.91 |
| 110% LRL | 0.69 (0.33-1.40) | 0.29 |  | 0.72 (0.33-1.55) | 0.40 |  | 0.70 (0.38-1.32) | 0.27 |
| 115% LRL | 0.58 (0.32-1.05) | 0.07 |  | 0.65 (0.31-1.33) | 0.24 |  | 1.01 (0.66-1.55) | 0.95 |
| 120% LRL | 0.40 (0.23-0.70) | <0.01 |  | 0.78 (0.43-1.41) | 0.41 |  | 0.97 (0.68-1.40) | 0.88 |
| GDM^a^ |  |  |  |  |  |  |  |  |
| 80% LRL | 0.50 (0.06-4.07) | 0.52 |  | 0.84 (0.25-2.89) | 0.78 |  | 12.57 (1.30-121.46) | 0.03 |
| 85% LRL | 0.32 (0.04-2.53) | 0.28 |  | 0.84 (0.35-2.04) | 0.71 |  | 12.57 (1.30-121.46) | 0.03 |
| 90% LRL | 0.37 (0.09-1.59) | 0.18 |  | 1.02 (0.51-2.04) | 0.97 |  | 1.65 (0.44-6.27) | 0.46 |
| 95% LRL | 0.49 (0.19-1.24) | 0.13 |  | 0.84 (0.46-1.53) | 0.57 |  | 1.03 (0.45-2.40) | 0.94 |
| 100% LRL | 0.27 (0.11-0.68) | 0.01 |  | 0.79 (0.47-1.35) | 0.39 |  | 1.47 (0.85-2.53) | 0.17 |
| 105% LRL | 0.28 (0.15-0.54) | <0.01 |  | 0.72 (0.46-1.15) | 0.17 |  | 1.09 (0.73-1.63) | 0.67 |
| 110% LRL | 0.35 (0.21-0.57) | <0.01 |  | 0.66 (0.43-1.00) | 0.05 |  | 0.88 (0.64-1.20) | 0.42 |
| 115% LRL | 0.41 (0.29-0.59) | <0.01 |  | 0.69 (0.48-1.01) | 0.06 |  | 0.92 (0.72-1.17) | 0.49 |
| 120% LRL | 0.41 (0.31-0.54) | <0.01 |  | 0.67 (0.48-0.94) | 0.02 |  | 0.87 (0.71-1.06) | 0.17 |
| ICP^a^ |  |  |  |  |  |  |  |  |
| 80% LRL | 0 | 0.99 |  | 0 | 0.99 |  | 51.58 (5.14-517.64) | <0.01 |
| 85% LRL | 0 | 0.99 |  | 4.17 (0.55-31.79) | 0.17 |  | 51.58 (5.14-517.64) | <0.01 |
| 90% LRL | 0 | 0.99 |  | 6.21 (1.42-27.27) | 0.02 |  | 15.65 (1.92-127.82) | 0.01 |
| 95% LRL | 0 | 0.99 |  | 3.87 (0.89-16.85) | 0.07 |  | 4.44 (0.58-33.92) | 0.15 |
| 100% LRL | 1.80 (0.24-13.55) | 0.57 |  | 4.48 (1.30-15.39) | 0.02 |  | 2.09 (0.28-15.73) | 0.47 |
| 105% LRL | 0.94 (0.13-7.04) | 0.95 |  | 3.16 (0.92-10.80) | 0.07 |  | 0.92 (0.12-6.89) | 0.94 |
| 110% LRL | 1.26 (0.29-5.44) | 0.76 |  | 2.44 (0.71-8.33) | 0.15 |  | 0.45 (0.06-3.36) | 0.44 |
| 115% LRL | 0.70 (0.16-3.00) | 0.63 |  | 1.91 (0.56-6.52) | 0.30 |  | 1.09 (0.37-3.22) | 0.88 |
| 120% LRL | 0.93 (0.31-2.76) | 0.89 |  | 1.47 (0.43-5.02) | 0.54 |  | 0.99 (0.39-2.54) | 0.98 |
| FGR^a^ |  |  |  |  |  |  |  |  |
| 80% LRL | 0 | 0.98 |  | 1.75 (0.23-13.16) | 0.59 |  | 10.77 (1.11-104.49) | 0.04 |
| 85% LRL | 0 | 0.99 |  | 0.89 (0.12-6.52) | 0.90 |  | 10.77 (1.11-104.49) | 0.04 |
| 90% LRL | 1.41 (0.19-10.55) | 0.74 |  | 0.61 (0.08-4.49) | 0.63 |  | 3.30 (0.42-26.02) | 0.26 |
| 95% LRL | 1.35 (0.32-5.64) | 0.68 |  | 1.66 (0.60-4.63) | 0.33 |  | 1.94 (0.46-8.19) | 0.37 |
| 100% LRL | 0.78 (0.19-3.23) | 0.74 |  | 1.53 (0.61-3.83) | 0.37 |  | 1.40 (0.43-4.51) | 0.58 |
| 105% LRL | 1.31 (0.56-3.03) | 0.53 |  | 1.31 (0.56-3.03) | 0.53 |  | 0.82 (0.30-2.26) | 0.70 |
| 110% LRL | 1.13 (0.54-2.37) | 0.74 |  | 1.19 (0.55-2.61) | 0.66 |  | 0.72 (0.33-1.58) | 0.42 |
| 115% LRL | 0.89 (0.47-1.68) | 0.72 |  | 1.24 (0.61-2.48) | 0.55 |  | 0.74 (0.41-1.34) | 0.32 |
| 120% LRL | 0.88 (0.52-1.50) | 0.64 |  | 1.07 (0.55-2.08) | 0.85 |  | 0.72 (0.44-1.17) | 0.18 |
| PA^a^ |  |  |  |  |  |  |  |  |
| 80% LRL | 0 | 0.98 |  | 0 | 0.98 |  | 0 | 0.99 |
| 85% LRL | 0 | 0.99 |  | 0 | 0.99 |  | 0 | 0.99 |
| 90% LRL | 0 | 0.98 |  | 0.89 (0.12-6.55) | 0.91 |  | 0 | 0.99 |
| 95% LRL | 0 | 0.98 |  | 0.54 (0.07-3.96) | 0.55 |  | 0 | 0.99 |
| 100% LRL | 0.54 (0.07-3.97) | 0.55 |  | 1.25 (0.39-4.04) | 0.71 |  | 0 | 0.98 |
| 105% LRL | 0.59 (0.14-2.41) | 0.46 |  | 0.88 (0.27-2.84) | 0.83 |  | 0 | 0.97 |
| 110% LRL | 0.56 (0.17-1.78) | 0.32 |  | 1.46 (0.62-3.41) | 0.38 |  | 0.28 (0.07-1.17) | 0.08 |
| 115% LRL | 0.53 (0.21-1.33) | 0.18 |  | 1.58 (0.75-3.34) | 0.23 |  | 0.47 (0.20-1.08) | 0.08 |
| 120% LRL | 0.61 (0.30-1.24) | 0.17 |  | 1.21 (0.57-2.55) | 0.62 |  | 0.50 (0.26-0.95) | 0.03 |
| PTB^a^ |  |  |  |  |  |  |  |  |
| 80% LRL | 0 | 0.98 |  | 0 | 0.98 |  | 3.34 (0.35-32.23) | 0.30 |
| 85% LRL | 0.80 (0.10-6.27) | 0.83 |  | 0.28 (0.04-2.07) | 0.21 |  | 3.34 (0.35-32.23) | 0.30 |
| 90% LRL | 1.44 (0.42-4.89) | 0.56 |  | 0.20 (0.03-1.42) | 0.11 |  | 2.33 (0.50-10.84) | 0.28 |
| 95% LRL | 1.44 (0.61-3.42) | 0.41 |  | 0.26 (0.06-1.05) | 0.06 |  | 2.08 (0.85-5.07) | 0.11 |
| 100% LRL | 1.10 (0.53-2.31) | 0.80 |  | 0.50 (0.19-1.19) | 0.11 |  | 1.29 (0.61-2.71) | 0.51 |
| 105% LRL | 1.02 (0.58-1.79) | 0.95 |  | 0.48 (0.22-1.03) | 0.06 |  | 1.07 (0.62-1.84) | 0.82 |
| 110% LRL | 0.85 (0.52-1.40) | 0.53 |  | 0.66 (0.36-1.20) | 0.17 |  | 1.06 (0.71-1.59) | 0.77 |
| 115% LRL | 0.76 (0.51-1.14) | 0.18 |  | 0.71 (0.42-1.19) | 0.19 |  | 1.08 (0.79-1.48) | 0.64 |
| 120% LRL | 0.80 (0.58-1.11) | 0.19 |  | 0.90 (0.59-1.38) | 0.63 |  | 1.15 (0.88-1.50) | 0.30 |
| PPH^a^ |  |  |  |  |  |  |  |  |
| 80% LRL | 1.26 (0.16-10.08) | 0.83 |  | 1.64 (0.48-5.60) | 0.43 |  | 0 | 0.98 |
| 85% LRL | 0.83 (0.11-6.44) | 0.86 |  | 0.82 (0.25-2.68) | 0.74 |  | 0 | 0.98 |
| 90% LRL | 0.91 (0.21-3.87) | 0.89 |  | 0.77 (0.28-2.15) | 0.62 |  | 2.23 (0.48-10.35) | 0.31 |
| 95% LRL | 2.04 (0.98-4.22) | 0.06 |  | 1.12 (0.56-2.26) | 0.75 |  | 1.62 (0.62-4.19) | 0.32 |
| 100% LRL | 1.42 (0.75-2.70) | 0.28 |  | 1.11 (0.60-2.03) | 0.75 |  | 1.69 (0.88-3.25) | 0.11 |
| 105% LRL | 1.32 (0.81-2.16) | 0.27 |  | 1.06 (0.62-1.80) | 0.83 |  | 1.22 (0.74-2.03) | 0.43 |
| 110% LRL | 1.14 (0.75-1.75) | 0.54 |  | 1.11 (0.69-1.77) | 0.67 |  | 1.29 (0.90-1.87) | 0.17 |
| 115% LRL | 1.36 (0.99-1.88) | 0.06 |  | 1.14 (0.75-1.73) | 0.53 |  | 1.29 (0.96-1.72) | 0.09 |
| 120% LRL | 1.30 (0.98-1.72) | 0.07 |  | 1.10 (0.75-1.60) | 0.64 |  | 1.16 (0.90-1.50) | 0.25 |
| Macrosomia^a^ | |  |  |  |  |  |  |  |
| 80% LRL | 1.85 (0.23-14.89) | 0.56 |  | 0.76 (0.10-5.67) | 0.79 |  | 4.96 (0.51-47.82) | 0.17 |
| 85% LRL | 2.71 (0.60-12.31) | 0.20 |  | 0.39 (0.05-2.88) | 0.36 |  | 4.96 (0.51-47.82) | 0.17 |
| 90% LRL | 1.36 (0.32-5.80) | 0.68 |  | 0.27 (0.04-1.99) | 0.20 |  | 5.66 (1.49-21.47) | 0.01 |
| 95% LRL | 1.24 (0.44-3.47) | 0.68 |  | 0.35 (0.08-1.41) | 0.14 |  | 3.04 (1.25-7.39) | 0.01 |
| 100% LRL | 1.75 (0.87-3.54) | 0.12 |  | 0.94 (0.43-2.05) | 0.88 |  | 2.07 (1.02-4.21) | 0.05 |
| 105% LRL | 1.21 (0.66-2.21) | 0.54 |  | 0.76 (0.37-1.57) | 0.46 |  | 1.42 (0.80-2.50) | 0.23 |
| 110% LRL | 1.10 (0.66-1.84) | 0.71 |  | 0.83 (0.44-1.55) | 0.56 |  | 1.38 (0.90-2.11) | 0.14 |
| 115% LRL | 0.84 (0.54-1.32) | 0.45 |  | 0.98 (0.58-1.66) | 0.94 |  | 1.28 (0.90-1.81) | 0.17 |
| 120% LRL | 0.79 (0.54-1.15) | 0.21 |  | 0.96 (0.60-1.55) | 0.88 |  | 1.00 (0.73-1.37) | 0.99 |
| ^a^: first trimester; OR: odds ratio; CI: confidence interval; LRL: lower reference interval limit; PE: preeclampsia; GH: gestational hypertension; GDM: gestational diabetes mellitus; ICP: intrahepatic cholestasis of pregnancy; FGR: fetal growth restriction; PA: placental abruption; PTB: preterm birth; PPH: postpartum hemorrhage; PS, protein S; PC, protein C; AT, antithrombin. | | | | | | | | |
|  |  |  |  |  |  |  |  |  |

| **eTable 3** Logistic regression analysis of the risk of PC, PS, and AT in the second trimester during singleton pregnancy for adverse perinatal outcomes | | | | | | | | |
| --- | --- | --- | --- | --- | --- | --- | --- | --- |
|  | PC | |  | PS | |  | AT | |
|  | OR (95% CI) | *p* value |  | OR (95% CI) | *p* value |  | OR (95% CI) | *p* value |
| FGR^b^ |  |  |  |  |  |  |  |  |
| 80% LRL | 2.35 (0.26-21.27) | 0.45 |  | 2.36 (0.26-21.35) | 0.44 |  | 0 | 0.99 |
| 85% LRL | 2.38 (0.50-11.35) | 0.28 |  | 2.75 (0.56-13.37) | 0.21 |  | 2.43 (0.27-21.97) | 0.43 |
| 90% LRL | 1.72 (0.38-7.88) | 0.48 |  | 1.70 (0.49-5.90) | 0.40 |  | 2.64 (0.73-9.60) | 0.14 |
| 95% LRL | 1.14 (0.34-3.83) | 0.84 |  | 1.25 (0.37-4.22) | 0.72 |  | 3.21 (1.48-6.99) | <0.01 |
| 100% LRL | 1.23 (0.47-3.17) | 0.67 |  | 0.92 (0.28-3.06) | 0.89 |  | 3.34 (1.96-5.69) | <0.01 |
| 105% LRL | 2.01 (1.07-3.76) | 0.03 |  | 1.20 (0.46-3.09) | 0.71 |  | 3.09 (1.96-4.87) | <0.01 |
| 110% LRL | 1.70 (0.99-2.92) | 0.05 |  | 1.32 (0.59-2.98) | 0.50 |  | 2.33 (1.57-3.45) | <0.01 |
| 115% LRL | 1.32 (0.80-2.17) | 0.28 |  | 1.47 (0.76-2.86) | 0.25 |  | 2.16 (1.48-3.15) | <0.01 |
| 120% LRL | 1.46 (0.95-2.26) | 0.09 |  | 1.21 (0.64-2.27) | 0.56 |  | 2.15 (1.47-3.14) | <0.01 |
| PA^b^ |  |  |  |  |  |  |  |  |
| 80% LRL | 0 | 0.99 |  | 0 | 0.99 |  | 0 | 0.99 |
| 85% LRL | 0 | 0.99 |  | 0 | 0.99 |  | 0 | 0.99 |
| 90% LRL | 0 | 0.99 |  | 0 | 0.99 |  | 0 | 0.99 |
| 95% LRL | 1.38 (0.18-10.49) | 0.76 |  | 1.50 (0.20-11.39) | 0.70 |  | 0 | 0.98 |
| 100% LRL | 0.86 (0.11-6.41) | 0.88 |  | 2.40 (0.55-10.46) | 0.24 |  | 1.84 (0.63-5.37) | 0.26 |
| 105% LRL | 0.46 (0.06-3.44) | 0.45 |  | 1.77 (0.41-7.62) | 0.44 |  | 1.36 (0.51-3.58) | 0.54 |
| 110% LRL | 0.28 (0.04-2.04) | 0.21 |  | 1.34 (0.31-5.75) | 0.69 |  | 1.09 (0.48-2.43) | 2.43 |
| 115% LRL | 0.18 (0.03-1.35) | 0.10 |  | 2.58 (0.97-6.86) | 0.06 |  | 1.30 (0.64-2.67) | 0.47 |
| 120% LRL | 0.40 (0.12-1.31) | 0.13 |  | 1.94 (0.73-5.13) | 0.18 |  | 1.43 (0.72-2.82) | 0.31 |
| PTB^b^ |  |  |  |  |  |  |  |  |
| 80% LRL | 1.6 (0.18-14.42) | 0.68 |  | 0 | 0.98 |  | 2.81 (0.25-31.27) | 0.40 |
| 85% LRL | 1.53 (0.32-7.27) | 0.60 |  | 1.63 (0.33-7.92) | 0.55 |  | 7.98 (1.32-48.28) | 0.02 |
| 90% LRL | 1.85 (0.50-6.82) | 0.36 |  | 0.61 (0.14-2.64) | 0.51 |  | 4.33 (1.48-12.67) | 0.01 |
| 95% LRL | 2.89 (1.28-6.52) | 0.01 |  | 0.46 (0.11-1.97) | 0.30 |  | 3.33 (1.66-6.69) | <0.01 |
| 100% LRL | 1.97 (0.98-3.98) | 0.06 |  | 0.53 (0.16-1.76) | 0.30 |  | 2.47 (1.51-4.04) | <0.01 |
| 105% LRL | 1.67 (0.95-2.93) | 0.08 |  | 0.40 (0.12-1.29) | 0.13 |  | 1.86 (1.21-2.85) | <0.01 |
| 110% LRL | 1.50 (0.94-2.40) | 0.09 |  | 0.53 (0.21-1.34) | 0.18 |  | 1.57 (1.12-2.22) | 0.01 |
| 115% LRL | 1.52 (1.01-2.27) | 0.04 |  | 0.77 (0.39-1.51) | 0.44 |  | 1.39 (1.01-1.93) | 0.04 |
| 120% LRL | 1.58 (1.10-2.26) | 0.01 |  | 0.70 (0.38-1.30) | 0.26 |  | 1.36 (1.00-1.85) | 0.05 |
| PPH^b^ |  |  |  |  |  |  |  |  |
| 80% LRL | 0 | 0.99 |  | 0 | 0.98 |  | 0 | 0.99 |
| 85% LRL | 0 | 0.99 |  | 0 | 0.99 |  | 2.67 (0.29-24.13) | 0.38 |
| 90% LRL | 0.99 (0.13-7.68) | 0.99 |  | 0 | 0.98 |  | 0.86 (0.11-6.61) | 0.88 |
| 95% LRL | 0.42 (0.06-3.13) | 0.40 |  | 0.44 (0.06-3.29) | 0.42 |  | 0.31 (0.04-2.29) | 0.25 |
| 100% LRL | 0.82 (0.25-2.71) | 0.75 |  | 0.69 (0.16-2.92) | 0.61 |  | 0.97 (0.44-2.16) | 0.94 |
| 105% LRL | 0.94 (0.40-2.23) | 0.89 |  | 0.52 (0.12-2.16) | 0.36 |  | 1.03 (0.55-1.93) | 0.94 |
| 110% LRL | 0.87 (0.46-1.76) | 0.69 |  | 0.61 (0.19-1.97) | 0.41 |  | 0.99 (0.61-1.61) | 0.97 |
| 115% LRL | 1.21 (0.70-2.08) | 0.50 |  | 0.87 (0.37-2.04) | 0.74 |  | 1.04 (0.67-1.60) | 0.86 |
| 120% LRL | 1.04 (0.63-1.72) | 0.87 |  | 0.65 (0.28-1.51) | 0.32 |  | 1.19 (0.80-1.77) | 0.40 |
| Macrosomia^b^ | |  |  |  |  |  |  |  |
| 80% LRL | 0 | 0.99 |  | 0 | 0.99 |  | 0 | 0.99 |
| 85% LRL | 0 | 0.99 |  | 0 | 0.99 |  | 0 | 0.99 |
| 90% LRL | 0 | 0.99 |  | 0 | 0.98 |  | 0 | 0.99 |
| 95% LRL | 0 | 0.98 |  | 0 | 0.98 |  | 0.57 (0.08-4.25) | 0.59 |
| 100% LRL | 0 | 0.98 |  | 0 | 0.98 |  | 0.70 (0.21-2.28) | 0.55 |
| 105% LRL | 0.25 (0.03-1.83) | 0.17 |  | 0.44 (0.06-3.26) | 0.42 |  | 0.56 (0.20-1.57) | 0.27 |
| 110% LRL | 0.82 (0.32-2.09) | 0.68 |  | 0.71 (0.17-2.99) | 0.64 |  | 0.52 (0.24-1.11) | 0.09 |
| 115% LRL | 0.63 (0.27-1.49) | 0.30 |  | 0.74 (0.23-2.42) | 0.62 |  | 0.50 (0.26-0.97) | 0.04 |
| 120% LRL | 0.60 (0.28-1.28) | 0.19 |  | 0.77 (0.27-2.17) | 0.62 |  | 0.55 (0.32-0.96) | 0.04 |
| ^b^: second trimester; OR: odds ratio; CI: confidence interval; LRL: lower reference interval limit; FGR: fetal growth restriction; PA: placental abruption; PTB: preterm birth; PPH: postpartum hemorrhage; PS, protein S; PC, protein C; AT, antithrombin. | | | | | | | | |
|  |  |  |  |  |  |  |  |  |

| **eTable 4** Logistic regression analysis of the risk of PC, PS, and AT in the third trimester during singleton pregnancy for adverse perinatal outcomes | | | | | | | | |
| --- | --- | --- | --- | --- | --- | --- | --- | --- |
|  | PC | |  | PS | |  | AT | |
|  | OR (95% CI) | *p* value |  | OR (95% CI) | *p* value |  | OR (95% CI) | *p* value |
| FGR^c^ |  |  |  |  |  |  |  |  |
| 80% LRL | 3.15 (0.57-17.33) | 0.19 |  | 3.18 (0.58-17.54) | 0.18 |  | 6.53 (0.41-105.00) | 0.19 |
| 85% LRL | 2.62 (0.50-13.64) | 0.25 |  | 1.76 (0.36-8.58) | 0.48 |  | 3.26 (0.29-36.15) | 0.34 |
| 90% LRL | 1.62 (0.34-7.68) | 0.55 |  | 1.36 (0.29-6.33) | 0.70 |  | 0.92 (0.11-7.51) | 0.94 |
| 95% LRL | 1.11 (0.32-3.83) | 0.87 |  | 1.36 (0.29-6.33) | 0.70 |  | 3.29 (1.39-7.80) | 0.01 |
| 100% LRL | 1.60 (0.65-3.97) | 0.31 |  | 1.07 (0.31-3.70) | 0.91 |  | 2.34 (1.12-4.90) | 0.02 |
| 105% LRL | 1.06 (0.47-2.40) | 0.88 |  | 1.10 (0.42-2.90) | 0.84 |  | 2.03 (1.09-3.77) | 0.03 |
| 110% LRL | 0.79 (0.35-1.75) | 0.55 |  | 0.75 (0.29-1.93) | 0.55 |  | 2.19 (1.35-3.58) | <0.01 |
| 115% LRL | 1.25 (0.72-2.19) | 0.43 |  | 0.64 (0.29-1.42) | 0.27 |  | 1.64 (1.09-2.47) | 0.02 |
| 120% LRL | 1.00 (0.61-1.62) | 0.99 |  | 0.59 (0.28-1.25) | 0.17 |  | 1.71 (1.22-2.39) | <0.01 |
| PA^c^ |  |  |  |  |  |  |  |  |
| 80% LRL | 0 | 0.99 |  | 0 | 0.99 |  | 0 | 0.99 |
| 85% LRL | 0 | 0.99 |  | 0 | 0.99 |  | 0 | 0.99 |
| 90% LRL | 0 | 0.99 |  | 0 | 0.99 |  | 0 | 0.99 |
| 95% LRL | 0 | 0.99 |  | 0 | 0.99 |  | 0 | 0.98 |
| 100% LRL | 0 | 0.98 |  | 2.67 (0.34-20.91) | 0.35 |  | 0 | 0.98 |
| 105% LRL | 0 | 0.98 |  | 1.62 (0.21-12.39) | 0.64 |  | 0.94 (0.13-7.10) | 0.95 |
| 110% LRL | 0 | 0.97 |  | 1.12 (0.15-8.52) | 0.91 |  | 1.13 (0.26-4.87) | 0.87 |
| 115% LRL | 0.54 (0.07-4.02) | 0.55 |  | 0.68 (0.09-5.11) | 0.71 |  | 1.73 (0.64-4.65) | 0.28 |
| 120% LRL | 0.69 (0.16-2.94) | 0.61 |  | 0.55 (0.07-4.15) | 0.56 |  | 0.84 (0.32-2.26) | 0.73 |
| PTB^c^ |  |  |  |  |  |  |  |  |
| 80% LRL | 3.52 (0.64-19.43) | 0.15 |  | 1.38 (0.16-11.89) | 0.77 |  | 6.78 (0.42-109.01) | 0.18 |
| 85% LRL | 2.69 (0.52-14.02) | 0.24 |  | 2.03 (0.42-9.87) | 0.38 |  | 3.38 (0.30-37.53) | 0.32 |
| 90% LRL | 1.69 (0.36-8.04) | 0.51 |  | 1.59 (0.34-7.45) | 0.55 |  | 2.26 (0.45-11.32) | 0.32 |
| 95% LRL | 2.33 (0.84-6.51) | 0.11 |  | 1.59 (0.34-7.45) | 0.55 |  | 2.88 (1.17-7.05) | 0.02 |
| 100% LRL | 1.74 (0.70-4.32) | 0.23 |  | 1.27 (0.37-4.37) | 0.71 |  | 2.90 (1.41-5.96) | <0.01 |
| 105% LRL | 1.13 (0.50-2.56) | 0.77 |  | 1.57 (0.64-3.87) | 0.33 |  | 2.36 (1.28-4.33) | 0.01 |
| 110% LRL | 0.99 (0.46-2.11) | 0.98 |  | 1.09 (0.46-2.63) | 0.84 |  | 1.72 (1.02-2.92) | 0.04 |
| 115% LRL | 1.05 (0.57-1.92) | 0.88 |  | 0.63 (0.27-1.48) | 0.29 |  | 1.90 (1.26-2.87) | <0.01 |
| 120% LRL | 1.16 (0.72-1.88) | 0.55 |  | 0.59 (0.27-1.30) | 0.19 |  | 1.28 (0.89-1.83) | 0.18 |
| PPH^c^ |  |  |  |  |  |  |  |  |
| 80% LRL | 0 | 0.98 |  | 0 | 0.98 |  | 0 | 0.99 |
| 85% LRL | 1.89 (0.22-15.92) | 0.56 |  | 0 | 0.99 |  | 5.80 (0.52-64.51) | 0.15 |
| 90% LRL | 2.90 (0.61-13.88) | 0.18 |  | 0 | 0.99 |  | 3.88 (0.77-19.55) | 0.10 |
| 95% LRL | 3.02 (0.99-9.22) | 0.05 |  | 0 | 0.99 |  | 1.05 (0.24-4.54) | 0.94 |
| 100% LRL | 3.04 (1.21-7.62) | 0.02 |  | 0.64 (0.09-4.87) | 0.67 |  | 1.38 (0.48-3.97) | 0.55 |
| 105% LRL | 2.73 (1.29-5.80) | 0.01 |  | 0.77 (0.18-3.25) | 0.72 |  | 1.28 (0.54-3.06) | 0.57 |
| 110% LRL | 2.62 (1.32-5.18) | 0.01 |  | 1.20 (0.42-3.42) | 0.74 |  | 1.20 (0.59-2.45) | 0.62 |
| 115% LRL | 1.89 (1.01-2.50) | 0.04 |  | 1.34 (0.60-3.00) | 0.48 |  | 1.64 (0.98-2.73) | 0.06 |
| 120% LRL | 1.53 (0.89-2.64) | 0.13 |  | 1.23 (0.58-2.62) | 0.59 |  | 1.32 (0.85-2.04) | 0.22 |
| Macrosomia^c^ | |  |  |  |  |  |  |  |
| 80% LRL | 6.76 (0.77-59.22) | 0.08 |  | 0 | 0.99 |  | 0 | 0.99 |
| 85% LRL | 13.98 (2.63-74.30) | <0.01 |  | 0 | 0.99 |  | 0 | 0.99 |
| 90% LRL | 8.69 (1.79-42.27) | 0.01 |  | 0 | 0.99 |  | 4.85 (0.58-40.32) | 0.14 |
| 95% LRL | 3.82 (0.86-17.03) | 0.08 |  | 0 | 0.99 |  | 1.46 (0.19-11.04) | 0.72 |
| 100% LRL | 2.44 (0.56-10.60) | 0.23 |  | 0 | 0.99 |  | 1.89 (0.44-8.11) | 0.39 |
| 105% LRL | 2.26 (0.67-7.58) | 0.19 |  | 0 | 0.98 |  | 2.52 (0.87-7.32) | 0.09 |
| 110% LRL | 1.71 (0.51-5.70) | 0.38 |  | 0 | 0.98 |  | 2.86 (1.24-6.63) | 0.01 |
| 115% LRL | 1.89 (0.73-4.93) | 0.19 |  | 0.94 (0.22-3.98) | 0.93 |  | 2.68 (1.32-5.44) | 0.01 |
| 120% LRL | 3.11 (1.53-6.31) | <0.01 |  | 1.18 (0.36-3.90) | 0.79 |  | 1.45 (0.73-2.86) | 0.29 |
| ^c^: third trimester; OR: odds ratio; CI: confidence interval; LRL: lower reference interval limit; FGR: fetal growth restriction; PA: placental abruption; PTB: preterm birth; PPH: postpartum hemorrhage; PS, protein S; PC, protein C; AT, antithrombin. | | | | | | | | |
|  |  |  |  |  |  |  |  |  |
